# Supplementary material for: Mechanisms governing the pioneering and redistribution capabilities of the non-classical pioneer PU.1
Source: Nat Commun. 2020 Jan 21;11:402. doi: 10.1038/s41467-019-13960-2 (PMC6972792; doi:10.1038/s41467-019-13960-2)
Supplement: Supplementary file 7 — Source data [file 41467_2019_13960_MOESM7_ESM.zip › Source_Data/Figure6/Figure6B_MotifScanOutput/ETS1_mutPU.1/homerResults/motif1.similar.html]

motif1

## Information for motif1

C
T
G
A
T
A
G
C
T
G
A
C
C
A
T
G
T
A
C
G
T
G
C
A
G
C
T
A
T
C
A
G
A
G
C
T
T
A
C
G
T
C
A
G
  
Reverse Opposite:  

A
G
T
C
A
T
G
C
T
C
G
A
A
G
T
C
C
G
A
T
A
C
G
T
A
T
G
C
G
T
A
C
A
C
T
G
A
T
C
G
G
A
C
T
  

|  |  |
| --- | --- |
| p-value: | 1e-3059 |
| log p-value: | -7.044e+03 |
| Information Content per bp: | 1.507 |
| Number of Target Sequences with motif | 10115.0 |
| Percentage of Target Sequences with motif | 75.01% |
| Number of Background Sequences with motif | 6221.7 |
| Percentage of Background Sequences with motif | 17.73% |
| Average Position of motif in Targets | 101.4 +/- 43.0bp |
| Average Position of motif in Background | 98.3 +/- 63.8bp |
| Strand Bias (log2 ratio + to - strand density) | 0.0 |
| Multiplicity (# of sites on avg that occur together) | 1.42 |
| Motif File: | file (matrix) reverse opposite |

### Similar de novo motifs found

|  |  |  |  |  |  |  |  |
| --- | --- | --- | --- | --- | --- | --- | --- |
| Rank | Match Score | Redundant Motif | P-value | log P-value | % of Targets | % of Background | Motif file |
| 1 | 0.982 | A G T C A T G C T C G A A G T C C G A T A C G T A T G C A G T C A C G T A T C G G A C T A G T C | 1e-2839 | -6538.853145 | 77.74% | 21.83% | motif file (matrix) |
| 2 | 0.980 | T C G A A G T C C G A T G C A T A T G C G T A C A C T G A T C G G A C T | 1e-2819 | -6492.602970 | 77.86% | 22.12% | motif file (matrix) |
| 3 | 0.959 | T C G A A G T C C G A T C G A T A G T C G T A C A C G T A T C G | 1e-2570 | -5919.947362 | 83.42% | 30.01% | motif file (matrix) |
| 4 | 0.930 | G T A C T A G C A G T C G A T C T C G A A G T C C G A T A C G T A G T C A G T C A C G T A T C G A G C T | 1e-2447 | -5634.756790 | 73.75% | 21.83% | motif file (matrix) |
| 5 | 0.862 | C T G A A G T C C G A T A C G T A G T C A G T C A C G T | 1e-2364 | -5445.018000 | 81.48% | 30.06% | motif file (matrix) |
| 6 | 0.863 | C T G A T C A G T C G A T A G C T G C A A C T G A C T G G T C A C G T A C T A G A G C T C A T G T C A G A C T G | 1e-2048 | -4717.771306 | 65.38% | 18.71% | motif file (matrix) |
| 7 | 0.939 | T G A C A T C G A C T G C G T A C G T A T C A G A G C T C T A G A C T G | 1e-1822 | -4197.139180 | 69.69% | 24.59% | motif file (matrix) |
| 8 | 0.790 | C T G A A G T C G T C A A C T G A C T G C G T A C G T A | 1e-1039 | -2394.660942 | 41.25% | 11.92% | motif file (matrix) |
| 9 | 0.887 | C T A G C T G A A T C G T G C A C T A G T C A G T C G A C T G A T A C G C A G T A T C G T A C G T A C G | 1e-999 | -2301.711630 | 31.73% | 6.74% | motif file (matrix) |
| 10 | 0.775 | C T G A A G T C G T C A C A T G A T C G C G T A C G T A C T G A | 1e-900 | -2074.152224 | 25.52% | 4.43% | motif file (matrix) |
| 11 | 0.821 | T G C A C T A G T A C G G T C A C G T A C T G A A G T C G A T C T C A G G A T C | 1e-555 | -1278.162624 | 34.23% | 13.30% | motif file (matrix) |
| 12 | 0.907 | A G T C C G A T A C G T A G T C A G T C A C T G A C T G | 1e-532 | -1226.848001 | 31.67% | 11.86% | motif file (matrix) |
| 13 | 0.873 | A G C T G C A T C G A T T A G C A G T C A C T G A T C G A G T C | 1e-457 | -1054.417828 | 23.43% | 7.55% | motif file (matrix) |
| 14 | 0.896 | A G T C T C A G A G T C A C G T A C G T A G T C A G T C A C T G A T C G A G T C A C T G A T G C A T G C A T C G | 1e-416 | -958.726800 | 22.24% | 7.36% | motif file (matrix) |
| 15 | 0.765 | C T G A A C G T A C G T A C G T G T A C G T A C A G C T A C T G A G C T | 1e-367 | -845.793781 | 15.96% | 4.30% | motif file (matrix) |
| 16 | 0.859 | C T G A T A G C G T A C A C T G C A T G C G T A C G T A T C A G C T A G T C A G | 1e-291 | -670.598538 | 17.59% | 6.23% | motif file (matrix) |
| 17 | 0.834 | T A G C T C G A T A G C A T G C A C T G A T C G C G T A C G T A G T C A A G T C T A G C T A C G T A C G | 1e-186 | -429.526525 | 8.65% | 2.38% | motif file (matrix) |
| 18 | 0.785 | G T A C A G T C A C T G A T C G C G T A G C A T T A G C A T G C T G C A | 1e-130 | -300.766149 | 8.03% | 2.77% | motif file (matrix) |
| 19 | 0.779 | A T C G A C T G C A T G A T C G A C G T A C G T A C G T A T G C G T A C A C T G A C T G C A G T | 1e-92 | -213.348503 | 4.66% | 1.36% | motif file (matrix) |
| 20 | 0.841 | G T A C T G A C A T C G C T A G C G T A C G T A A T G C G A C T | 1e-19 | -44.187983 | 3.06% | 1.69% | motif file (matrix) |
